# The Interaction Between lncRNA SNHG6 and hnRNPA1 Contributes to the Growth of Colorectal Cancer by Enhancing Aerobic Glycolysis Through the Regulation of Alternative Splicing of PKM

**DOI:** 10.3389/fonc.2020.00363

**Published:** 2020-03-31

**Authors:** Zhixian Lan, Xiang Yao, Kangyue Sun, Aimin Li, Side Liu, Xinke Wang

**Affiliations:** Guangdong Provincial Key Laboratory of Gastroenterology, Department of Gastroenterology, Nanfang Hospital, Southern Medical University, Guangzhou, China

**Keywords:** colorectal cancer, small nucleolar RNA host gene 6, comprehensive RNA-binding proteins–mass spectrometry, bioinformatics analysis, heterogeneous nuclear ribonucleoprotein A1, pyruvate kinase M, metabolism

## Abstract

**Background:** Small nucleolar RNA host gene 6 (SNHG6) acts as a carcinogenic gene in colorectal cancer (CRC). However, previous studies on the mechanism by which long non-coding RNA (lncRNA) SNHG6 exerts its carcinogenic effect in CRC have not involved the direct interaction between SNHG6 and proteins, which is a very important carcinogenic mechanism of lncRNAs. Hence, our study conducted a comprehensive RNA-binding proteins–mass spectrometry (ChIRP–MS) analysis on SNHG6 to further explore its carcinogenic mechanism in CRC.

**Methods:** Proteins that interact with SNHG6 were found using ChIRP–MS analysis and were used to construct the protein–protein interactive (PPI) network using STRING, while the core module of the PPI network was identified using the MCODE plugin in Cytoscape. Pathway enrichment analyses, using WebGestalt, were performed on proteins and RNAs that were found to be associated with the expression of SNHG6 or which directly interacted with SNHG6. Finally, CatRAPID, miRbase, and TargetScanHuman were used to identify the sites of interaction between SNHG6, heterogeneous nuclear ribonucleoprotein A1 (hnRNPA1), and pyruvate kinase M (PKM) mRNA.

**Results:** The expression of SNHG6 in CRC was found to be higher than that of normal tissues and was positively correlated with a poor prognosis (*p* < 0.05). A total of 467 proteins that are able to interact with SNHG6 in CRC cells were identified using ChIRP–MS analysis and were used to create a PPI network, within which a core module composed of 44 proteins that performed the function of splicing mRNA, including hnRNPA1, was found to be positively correlated with SNHG6 (*p* < 0.05). The results of the pathway enrichment analyses suggested that SNHG6 played an important role in the metabolism of CRC by affecting the expression of PKM and SNHG6. The increase in the ratio of PKM2/PKM1 was proven using quantitative real-time polymerase chain reaction analysis. Further exploration suggested that SNHG6 could bind to hnRNPA1 and PKM.

**Conclusion:** SNHG6 was found to be able to target the mRNA of PKM as well as induce hnRNPA1 to specifically splice PKM mRNA, which increased the proportion of PKM2/PKM1, which may be an important carcinogenic mechanism in CRC that proceeds through the enhancement of aerobic glycolysis in CRC cells.

## Introduction

CRC is one of the most common cancers with an extremely high rate of cancer-associated mortality (1, 2). The frequency of recurrence and metastasis in patients with CRC is high, even in patients who have undergone surgical resection, which leads to a poor prognosis of CRC (3). Therefore, there is an urgent need to study the deep molecular mechanism that undermines the occurrence and development of CRC.

Long non-coding RNA (lncRNA) are functionally defined as transcripts with a length of more than 200 nucleotides. Small nucleolar RNA host gene 6 (SNHG6) is located on chromosome 8q13, a region with numerous copy number amplification in CRC (4), and encoding two ncRNAs: U87 C/D box snoRNA (SNORD87) (5) and lncRNA SNHG6. It has been reported that SNHG6 has been found to function as an oncogene in various cancers, including CRC, hepatocellular carcinoma, and breast cancer (6–8). Some studies have revealed that SNHG6 was found to be closely associated with cell proliferation, migration, pathological grade, and lymph node metastasis in CRC (6, 9, 10).

Alternative splicing (AS) is a common mechanism by which gene expression is regulated in cancers (11, 12). Heterogeneous nuclear ribonucleoproteins are one of the most important and classical AS regulators. The regulation of pyruvate kinase M (PKM) precursor mRNA splicing by hnRNPA1 is an important example (13–15). The PKM gene encodes for a primary transcript that contains two mutually exclusive exons, exon 9 and exon 10. The binding of hnRNPA1 to the splice sites flanking exon 9 in PKM transcripts resulted in exon 9 exclusion and exon 10 inclusion to generate PKM2 (13, 14). PKM1 is mainly expressed in normal human tissues, and an increase in the proportion of PKM2/PKM1 is often found in cancers, which could partially explain the Warburg effect observed in cancers, since PKM2 increases tumor cell sensitivity toward aerobic glycolysis (16, 17).

At present, SNHG6 in CRC exerts its carcinogenic effect by competing with endogenous RNA to sponge microRNAs (6, 9, 18). However, SNHG6 can interact not only with microRNAs but also with proteins to promote the proliferation and metastasis of CRC. lncRNAs can be used as a scaffold to draw two or more proteins into a complex (19) or as a guide to recruit proteins, such as variable splicing protein, to mRNAs (20, 21). As a result, we conducted a comprehensive RNA-binding proteins–mass spectrometry (ChIRP–MS) analysis to identify proteins that interact with SNHG6 to further explore the carcinogenic mechanism of SNHG6 in CRC.

**Figure 1:**
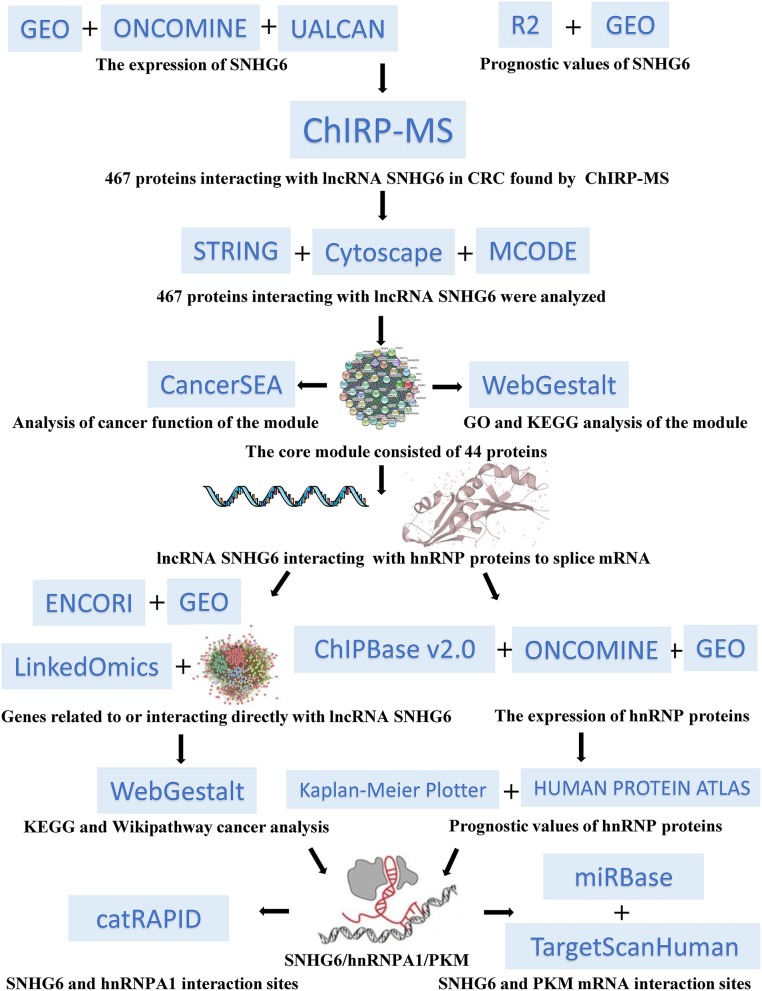
Overall workflow chart for this research study.

In our study (Figure 1), we found that the high expression of SNHG6 in CRC was associated with a poor prognosis in CRC patients. The results of the ChIRP–MS analysis suggested that SNHG6 could interact with hnRNP proteins, suggesting that SNHG6 may be involved in the splicing or processing of mRNA. The enrichment analysis conducted using data in Gene Expression Omnibus (GEO) and other bioinformatics databases found that SNHG6 may be involved in the metabolic processes of CRC and may target the precursor mRNA of PKM. Given that hnRNPA1 could perform variable splicing of the precursor mRNA of PKM to increase the proportion of PKM2/PKM1 (13, 14, 16), we assumed that it was possible for SNHG6 to interact with hnRNPA1 as well as induce hnRNPA1 to target the precursor mRNA of PKM and participate in the variable splicing process, which would promote the occurrence and the development of CRC.

## Methods

### Expression and Prognostic Values of SNHG6 in Colorectal Cancer

#### Expression of SNHG6 in Colorectal Cancer

AnnoLnc is a web server used to analyze novel human lncRNAs, which can be utilized to compare the expression levels of SNHG6 in different tumors (22). UALCAN is an interactive web portal used to perform in-depth analyses of The Cancer Genome Atlas (TCGA) gene expression data (23). Oncomine 4.5 is an online platform that provides data from cancer microarray datasets and can be used for data mining in multiple cancers (24). UALCAN and Oncomine 4.5 are both used to evaluate the expression of SNHG6 in CRC. We also used GSE81861 colorectal single-cell expression data from GEO and Cancer Single-Cell State Atlas (CancerSEA), the first dedicated database decoding distinct functional states of cancer cells at single-cell resolution (25), to explore the expression characteristics of SNHG6 at the single-cell level. The lncLocator is a subcellular localization predictor of lncRNAs based on a stacked ensemble classifier, which can be used to identify the distribution of SNHG6 in CRC cells (26).

#### Prognostic Value of SNHG6 in Colorectal Cancer

The prognostic value of SNHG6 in CRC was determined by analyzing the data of 177 CRC patients from GSE17538. The analysis was performed as previously described (10). In addition, the R2: Genomics Analysis and Visualization Platform, which is a web-based genomics analysis and visualization application, was also used (27).

### Comprehensive RNA-Binding Proteins–Mass Spectrometry Analysis

ChIRP–MS analysis is the most suitable method to detect target proteins directly controlled by RNAs. Therefore, we used ChIRP–MS to identify proteins that interact with SNHG6 in CRC (for more information, see the Supplementary Material: ChIRP–MS Analysis Method).

### Bioinformatics Analysis of Comprehensive RNA-Binding Proteins–Mass Spectrometry Results

#### Protein–Protein Interactive Network and Module Identification

String 11.0, a database used to determine the integrated functions of multiple genes (28), was used to build the protein–protein interactive (PPI) network with the highest confidence (0.900) by inputting proteins that interact with SNHG6 obtained through the results of the ChIRP–MS analysis. Cytoscape 3.7.1 is an open-source software platform used for visualizing complex networks (29, 30). The MCODE plugin in Cytoscape 3.7.1 was used to identify the most densely connected region in the PPI based on topology with the two k-cores, which could identify core modules and hub genes in the PPI network (31).

#### Functional Enrichment Analysis of 44 Genes in the Module

CancerSEA was used to explore whether the module identified using the MCODE plugin in Cytoscape 3.7.1 was associated with the carcinogenic process. A *P* < 0.05 was considered to be statistically significant. Gene ontology (GO) and the Kyoto Encyclopedia of Genes and Genomes (KEGG) enrichment analyses of genes in the module were performed using WebGestalt, a functional enrichment analysis web tool (32). An adjusted *P* < 0.05 was considered to be statistically significant.

#### Characteristics of hnRNP Proteins in Colorectal Cancer

##### Co-expression analysis of hnRNP proteins and SHNG6 in colorectal cancer

The co-relationship between hnRNP proteins and SHNG6 in CRC was evaluated using ChIPBase v2.0, which is an open database used for studying the transcriptional regulatory networks of lncRNAs, miRNAs, and protein-coding genes identified using ChIP-seq data (33). A *P* < 0.05 was considered to indicate statistical significance.

##### Expression of hnRNP proteins in colorectal cancer

The experimental data of Notterman and Alon colon statistics in Oncomine were used to evaluate the expression characteristics of hnRNP proteins in CRC, compared with that of colon tissues. A *P* < 0.05 was considered to indicate statistical significance.

##### Prognostic value of hnRNP proteins in colorectal cancer

The prognostic value of hnRNP proteins in CRC was analyzed using the Kaplan–Meier plotter database, which is capable of assessing the effect of 54 k genes on the survival of patients in 21 cancer types (34), as well as The Human Protein Atlas, which contains detailed information on proteins found in over 17 types of tumors and normal human cells (35). A *P* < 0.05 was considered to be statistically significant.

##### Expression of hnRNP proteins in drug-resistant colorectal cancer cell lines

The expression of hnRNPA1 in drug-resistant CRC cell lines was examined using data from GSE118490 and GSE11440. A *P* < 0.05 was considered to be statistically significant.

### Pathway Enrichment Analysis of SNHG6 in Colorectal Cancer

#### Pathway Enrichment Analysis of 467 Proteins of SNHG6 in Colorectal Cancer

KEGG and Wikipathway cancer enrichment analyses of genes in SNHG6 obtained through the ChIRP–MS analysis were performed in WebGestalt. A *P* < 0.05 was considered to be statistically significant.

#### Pathway Enrichment Analysis of Genes Associated With SNHG6 Expression in Colorectal Cancer

LinkedOmics, an open platform used for analyzing 32 TCGA cancer-associated datasets, was used to identify differentially expressed genes which are correlated with SNHG6 in the TCGA CRC cohort (36). The results were statistically analyzed using Pearson's correlation coefficient, followed by KEGG and Wikipathway cancer enrichment analyses conducted in WebGestalt, using the results obtained from the LinkedOmics platform. A *P* < 0.05 was considered to be statistically significant.

#### Pathway Enrichment Analysis of mRNA Interacting Directly With SNHG6

The interaction between mRNA–SNHG6 pairs was evaluated using The Encyclopedia of RNA Interactomes (ENCORI), which is an open-source web tool used for studying the ncRNA interactions of CLIP-seq, degradome-seq, and RNA–RNA interactome data (37). A *P* < 0.05 was considered to be statistically significant.

KEGG and Wikipathway cancer enrichment analyses were conducted in WebGestalt using the results from ENCORI. An adjusted *P* < 0.05 was considered to be statistically significant.

#### Pathway Enrichment Analysis of Genes Correlated With SNHG6 in GSE103479

The gene expression profiles of 156 colorectal cancer samples were downloaded from GSE103479. Based on the SNHG6 expression level, the top 3% and the bottom 3% of the samples were grouped as the high SNHG6 group and low SNHG6 group, respectively. The limma package in R was used to identify the differentially expressed genes between the two groups using cutoff values of |logFC| > 2.5 and *p* < 0.01. WebGestalt was used to explore the KEGG and Wikipathway cancer enrichment pathways based on the differentially expressed genes. An adjusted *P* < 0.05 was considered to be statistically significant.

### Quantitative Real-Time Polymerase Chain Reaction

Total RNA extraction, reverse transcription, and quantitative real-time polymerase chain reaction (qRT-PCR) were performed as previously described (10). The sequences of the primers used were as follows:

**Table:** 

PKM1 mRNA (sense):	5′-CGAGCCTCAAGTCACTCCAC -3′,
PKM1 mRNA (antisense):	5′-GTGAGCAGACCTGCCAGACT -3′,
PKM2 mRNA (sense):	5′-ATTATTTGAGGAACTCCGCCGCCT -3′,
PKM2 mRNA (antisense):	5′-ATTCCGGGTCACAGCAATGATGG -3′,
SNHG6 mRNA (sense):	5′-TTGAGGTGAAGGTGTATG -3′,
SNHG6 mRNA (antisense):	5′-GGTAACGAAGCAGAAGTA -3′,
GAPDH (sense):	5′-GATATTGTTGCCATCAATGAC-3′ and
GAPDH (antisense):	5′-TTGATTTTGGAGGGATCTCG-3′.

### Associations in the SNHG6/hnRNPA1/PKM Axis

#### The Binding Site Between SNHG6 and hnRNPA1

CatRAPID, an algorithm used to estimate the binding propensity of protein–RNA pairs (38), was used to predict the binding site between SNHG6 and hnRNPA1 by combining the secondary structure, hydrogen bonding, and van der Waals contributions.

#### Splicing Regulatory Motif of SNHG6 Combined With hnRNPA1

RegRNA 2.0, an integrated web server used for identifying functional RNA motifs in an input RNA sequence (39), was used to predict the splicing regulatory motif of SNHG6.

#### Functional Domains of hnRNPA1 Associated With SNHG6

Pfam is a database of 17,929 protein families (40), while the Simple Modular Architecture Research Tool (SMART) is a web database used for the identification of protein domains and the analysis of protein domain architectures (41). Both of these tools were used to identify the functional domains of hnRNPA1 associated with SNHG6.

#### Exploration of the Targeted mRNA of SNHG6

MicroRNAs homologous to SNHG6 were identified using the miRBase database (42). Thereafter, the TargetScanHuman database was searched to identify potential target genes of the microRNAs homologous to SNHG6 (43), which may also be the target genes of SNHG6 (44).

## Results

### Expression and Prognostic Values of SNHG6 in Colorectal Cancer

#### High Expression of SNHG6 in Colorectal Cancer

**Figure 2:**
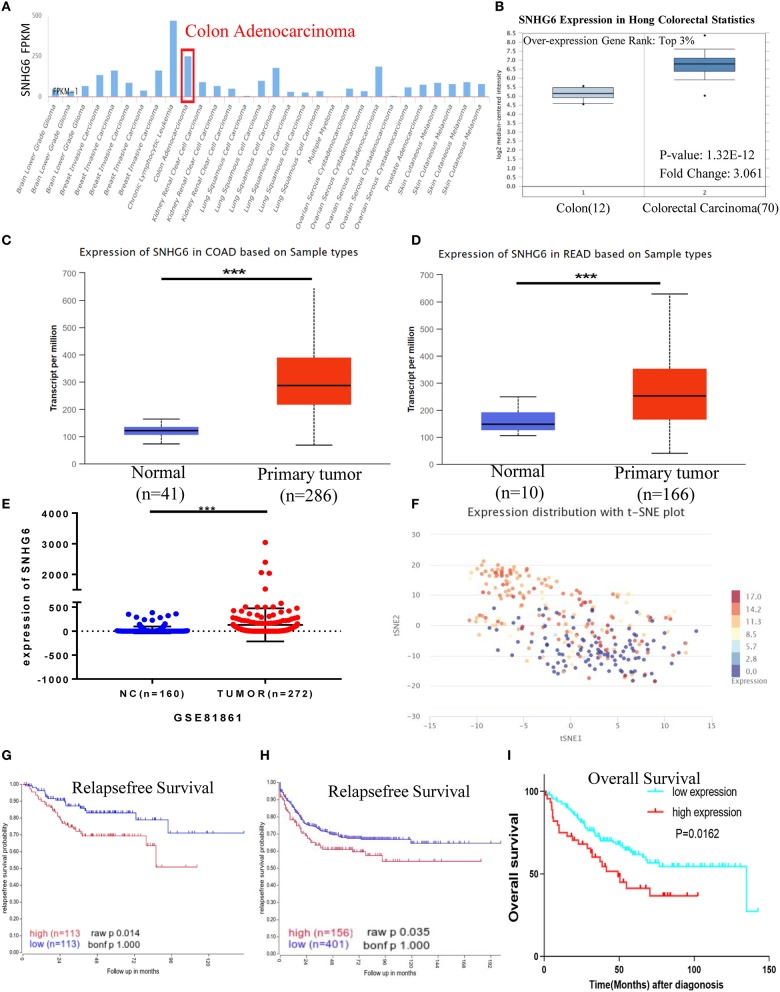
Expression and prognostic value of SNHG6 in colorectal cancer (CRC). **(A)** The expression of SNHG6 in human tumors. **(B–E)** High expression of SNHG6 in CRC tissues, compared with normal tissues. **(F)** The expression of SNHG6 in CRC cells was found to be heterogeneous. **(G–I)** The high expression of SNHG6 was found to be associated with poor prognosis in patients with CRC. Ns, *P* > 0.05; ^*^P < 0.05; ^**^P < 0.01; ^***^P < 0.001; ^****^P < 0.0001.

Data in AnnoLnc showed that the expression of SNHG6 in CRC was relatively high compared with that of many other tumors (Figure 2A), while SNHG6 was found to be mainly distributed in the cytoplasm of CRC cells based on the lncLocator and part of the nucleus (6). The differential expression of SNHG6 between CRC and normal tissues was analyzed using ULCAN (10) and Hong statistics in Oncomine, which found a relatively high expression of SNHG6 in CRC that was statistically different (Figures 2B–D). GSE81861, which contains single-cell sequencing data of CRC, was obtained from the GEO database to determine the SNHG6 expression in 272 CRC single cells and 160 normal colorectal cells, which found that SNHG6 was highly expressed in CRC (Figure 2E). Cancersea was also used to explore the clustering of CRC single cells based on the expression of SNHG6, which indicates the heterogeneous expression of SNHG6 in different CRC cells (Figure 2F).

#### High Expression of SNHG6 Is Associated With Poor Prognosis in Colorectal Cancer

The relapse-free survival (RS) of SNHG6 was analyzed in 226 and 557 CRC patients in GSE14333 and GSE39582, respectively. The results indicated that a high expression of SNHG6 was associated with a statistically significant higher recurrence rate in CRC patients (*p* = 0.014 and *p* = 0.035, respectively) (Figures 2G,H). The data of 177 CRC patients in GSE17538 was also used to analyze overall survival (OS), which showed that the OS rate was statistically lower when the expression of SNHG6 was high (*p* = 0.0162) (Figure 2I) (10).

### 467 Proteins Interact With SNHG6 in Colorectal Cancer

A total of 467 proteins were found to interact with SNHG6 (for details, see the Supplementary Material: Results of ChIRP–MS).

### Bioinformatics Analysis of Comprehensive RNA-Binding Proteins–Mass Spectrometry Results

#### Protein–Protein Interactive Network and Module Identification

**Figure 3:**
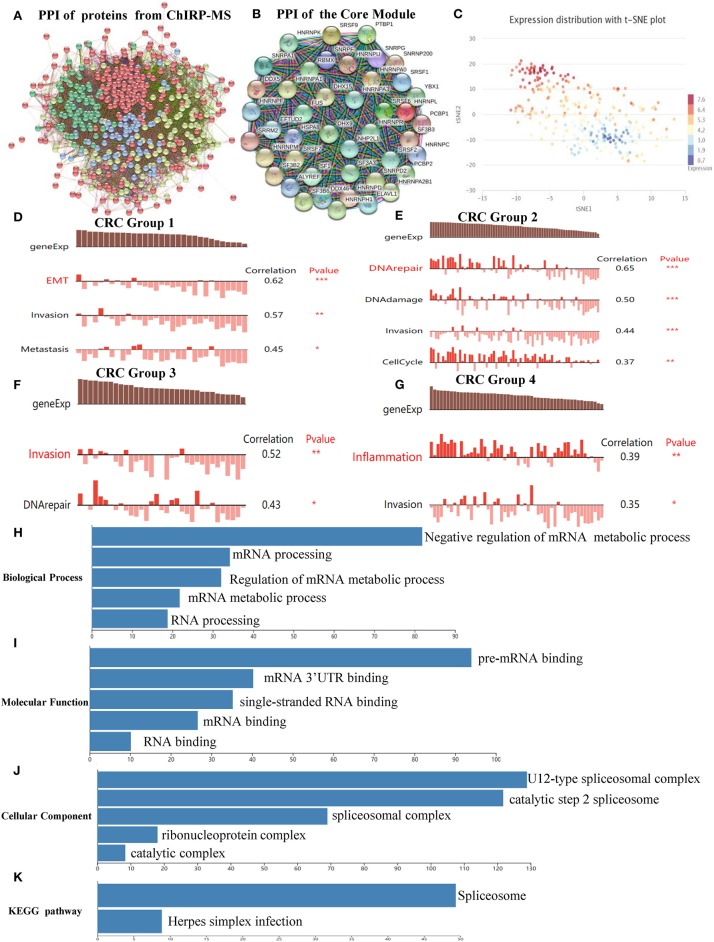
Protein–protein interactive (PPI) network and core module analysis. **(A)** The PPI consisted of 461 nodes with 5,226 edges. **(B)** The core module consisted of 44 nodes with 44 scores. **(C)** The overall expression of the module in colorectal cancer was found to be heterogeneous. **(D–G)** Carcinogenic process in different cell subsets from the core module. **(H–K)** The analysis of biological processes, molecular functions, cellular components, and Kyoto Encyclopedia of Genes and Genomes of the core module showed that it could participate in the splicing and the processing of precursor mRNA. Ns, *P* > 0.05; ^*^P < 0.05; ^**^P < 0.01; ^***^P < 0.001; ^****^P < 0.0001.

The PPI network, which comprised of 461 nodes with 5,226 edges, was constructed by importing 467 proteins specifically associated with SNHG6, found through the ChIRP–MS analysis, into STRING11.0 (Figure 3A). Each protein can be associated with various other proteins, which could result in collaborative modules that partake in the regulation of SNHG6 in CRC. As a result, we utilized the MCODE plugin in Cytoscape 3.7.1 to identify core modules within the PPI and then identified the core module with the highest score, which consisted of 44 nodes with 44 scores (Figure 3B) and was mainly composed of proteins that performed mRNA splicing, such as the hnRNP protein family.

#### The Module Could Promote Colorectal Cancer by Splicing and Processing mRNA

According to the clustering results of CancerSEA, the overall expression of the module in CRC was found to be heterogeneous (Figure 3C). Therefore, CancerSEA was used to determine whether the module was related to the carcinogenic process in different CRC cell subsets, and four groups with carcinogenic function were found. The results showed that Group 1 was correlated with epithelial–mesenehymal transition (EMT), invasion, and metastasis (Figure 3D); Group 2 was correlated with DNA repair, DNA damage, invasion, and cell cycle (Figure 3E); Group 3 was correlated with invasion and DNA repair (Figure 3F); and Group 4 was correlated with inflammation and invasion (Figure 3G).

In order to further investigate the mechanism by which the module could promote cancer, GO and KEGG analyses were conducted in WebGestalt. The top five terms of biological processes (BP), molecular functions (MF), cellular components (CC) as well as KEGG were enriched with a false discovery rate of <0.05. The top three terms of BP that were enriched were related to the negative regulation of the mRNA metabolic process, mRNA processing, and regulation of mRNA metabolic processes (Figure 3H), which are mainly focused on mRNA regulation and metabolic processes. The top three terms of MF that were enriched were related to pre-mRNA binding, mRNA 3'-UTR binding, and single-stranded RNA binding (Figure 3I). The top three terms of CC that were enriched were related to U12-type splicesomal complex, catalytic step 2 spliceosome, and splicesomal complex (Figure 3J). The top terms of KEGG that were enriched were related to spliceosomes (Figure 3K). Based on the above enrichment results of BP, MF, CC, and KEGG, we speculated that the main function of the proteins in the module was to participate in the splicing and the processing of precursor mRNA, which in turn affected the expression of genes that promoted the occurrence and the development of CRC.

#### Characteristics of hnRNP Proteins in Colorectal Cancer

**Figure 4:**
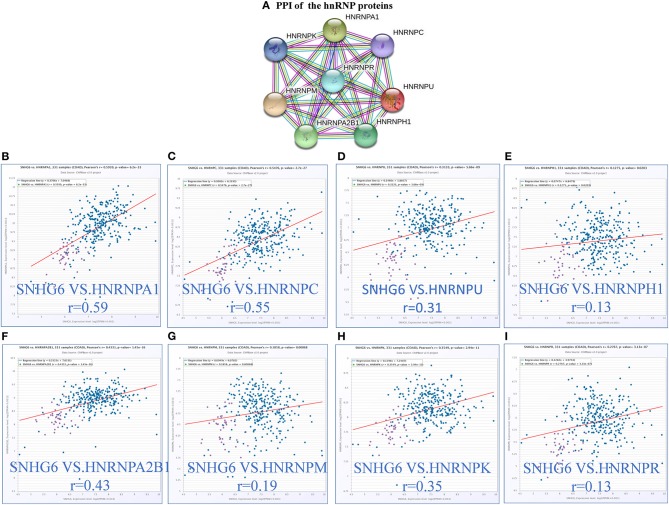
The expression of hnRNP proteins was positively correlated with SNHG6 in colorectal cancer (CRC). **(A)** The first eight hnRNP proteins with the strongest interaction with SNHG6 in the module. **(B–I)** The expression of hnRNP proteins was found to be positively correlated with SNHG6 in CRC.

According to the results of the core module obtained from the ChIRP–MS analysis, six of the top 10 proteins with the strongest level of interaction with SNHG6 belonged to the hnRNP protein family. Therefore, we assumed that the hnRNP proteins play an important role in the module and are closely related to the carcinogenic mechanism of SNHG6. Therefore, we identified the first eight hnRNP proteins in the module with the strongest levels of interaction with SNHG6 for further analysis: hnRNPA1, hnRNPC, hnRNPU, hnRNPH1, hnRNPA2B1, hnRNPM, hnRNPK, and hnRNPR (Figure 4A).

##### The expression of hnRNP proteins was positively correlated with SNHG6 expression in colorectal cancer

The correlation between the hnRNP proteins and SHNG6 in CRC was evaluated using ChIPBase v2.0, and all of the hnRNP proteins were found to be positively correlated with SNHG6 with a *P* > 0.05 (Figures 4B–I).

##### High expression of hnRNP proteins in colorectal cancer

**Figure 5:**
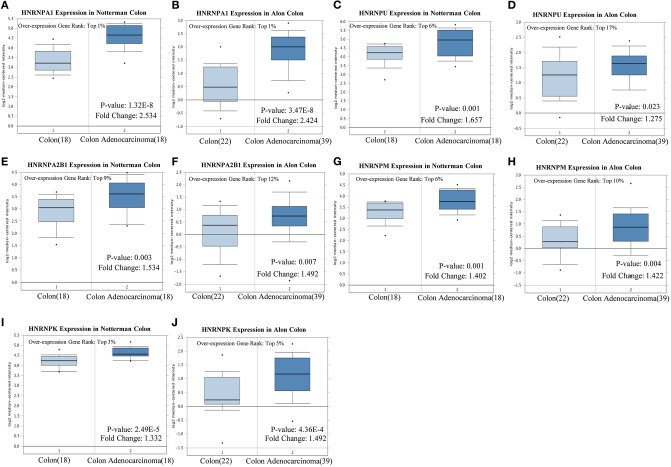
High expression of hnRNP proteins in colorectal cancer (CRC). **(A–J)** hnRNPA1, hnRNPU, hnRNPA2B1, hnRNPM, and hnRNPK were found to be highly expressed in CRC, with a statistical difference, based on both the Notterman and the Alon experimental data.

Notterman and Alon colon statistics in Oncomine were used to evaluate the expression of hnRNPA1, hnRNPC, hnRNPU, hnRNPH1, hnRNPA2B1, hnRNPM, hnRNPK, and hnRNPR in CRC compared with that of colon tissues, and only hnRNPA1, hnRNPU, hnRNPA2B1, hnRNPM, and hnRNPK were found to be highly expressed in CRC with significant statistical differences based on both Notterman and Alon experimental data (Figures 5A–J).

##### Low expression of hnRNP proteins was associated with poor prognosis in colorectal cancer

**Figure 6:**
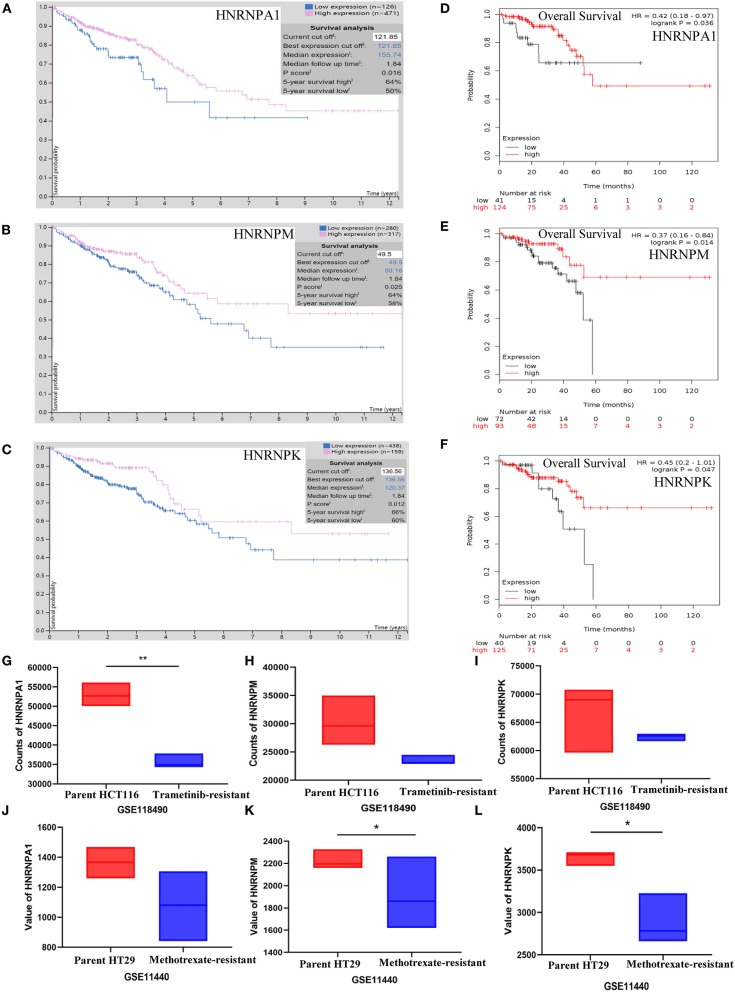
Correlation between the expression of hnRNP proteins and prognosis of colorectal cancer (CRC) patients. **(A–F)** Low expression of hnRNPA1, hnRNPM, and hnRNPK was found to be associated with poor prognosis in CRC. **(G–L)** The expression of hnRNPA1, hnRNPM, and hnRNPK was relatively low in drug-resistant CRC cells. Ns, *P* > 0.05; ^*^P < 0.05; ^**^P < 0.01; ^***^P < 0.001; ^****^P < 0.0001.

The prognostic value of hnRNPA1, hnRNPU, hnRNPA2B1, hnRNPM, and hnRNPK in CRC was analyzed using the Kaplan–Meier plotter and The Human Protein Atlas. The results were consistent with the fact that the high expression of hnRNP proteins was found to be related with a better prognosis, but only the results of hnRNPA1, hnRNPM, and hnRNPK were statistically significant in both the Kaplan–Meier plotter (Figures 6A–C) and The Human Protein Atlas (Figures 6D–F).

##### Relatively low expression of hnRNPA1, hnRNPM, and hnRNPK in drug-resistant colorectal cells

The survival analysis above showed that the low level of expression of hnRNPA1, hnRNPM, and hnRNPK was associated with poor prognosis, which was confusing. Therefore, we explored the expression of hnRNPA1, hnRNPM, and hnRNPK in drug-resistant CRC cells by analyzing GSE118490 and GSE11440, which showed that the expression of hnRNPA1, hnRNPM, and hnRNPK in drug-resistant CRC cells was relatively low (Figures 6G–L), which could reasonably explain the results of the survival curve.

### SNHG6 Plays an Important Role in the Metabolism of Colorectal Cancer

From the analysis given above, we speculated that SNHG6 may promote the occurrence of CRC by interacting with certain hnRNP proteins and affect the splicing of mRNA. However, it is not known what type of mRNA functioned as the clipped object. Therefore, we needed to identify SNHG6-related shearing objects and explore the process by which SNHG6 played a carcinogenic role.

#### Pathway Enrichment Analysis of 467 Proteins Obtained From the Comprehensive RNA-Binding Proteins–Mass Spectrometry Analysis of Colorectal Cancer

**Figure 7:**
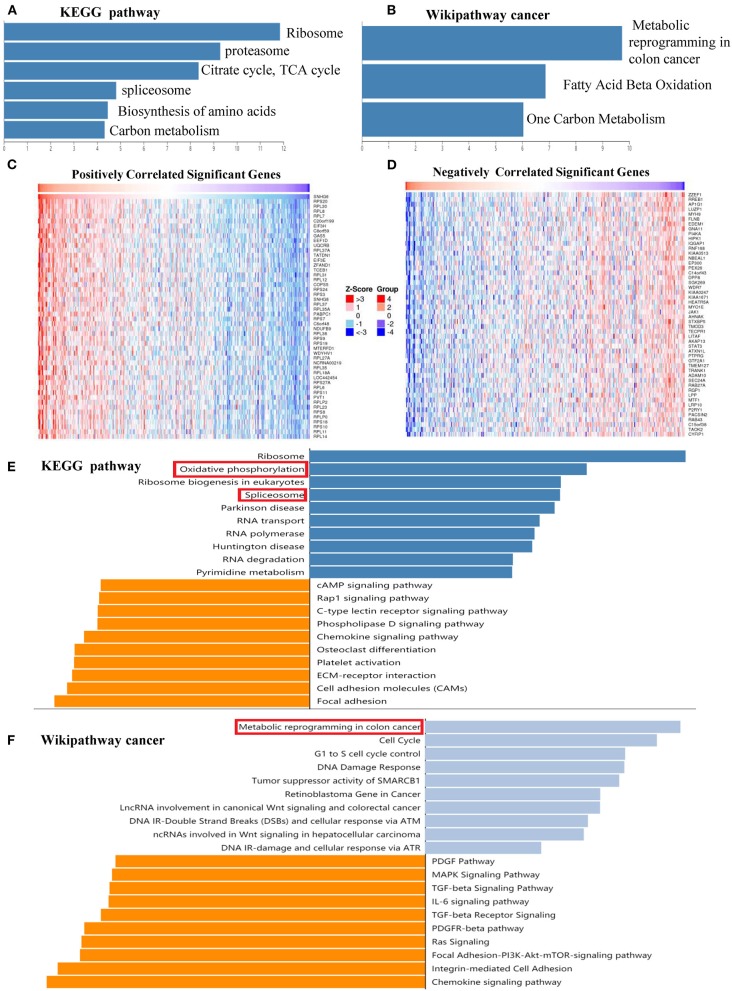
SNHG6 plays an important role in the metabolism of colorectal cancer (CRC). **(A,B)** Kyoto Encyclopedia of Genes and Genomes (KEGG) and Wikipathway cancer enrichment analysis of genes conducted on SNHG6 using comprehensive RNA-binding proteins–mass spectrometry. **(C,D)** Genes that were positively or negatively related to SNHG6 in CRC, as given by LinkedOmics. **(E,F)** KEGG and Wikipathway cancer enrichment analysis of genes that were positively or negatively related to SNHG6.

KEGG and Wikipathway cancer enrichment analyses of genes identified in the SNHG6 ChIRP–MS analaysis were conducted in WebGestalt. The terms for ribosome, proteasome, citrate cycle, spliceosome, biosynthesis of amino acids, and carbon metabolism were enriched in the KEGG enrichment analysis (Figure 7A). The terms for metabolic reprogramming in colon cancer, fatty acid beta oxidation, and one carbon metabolism were enriched in the Wikipathway cancer enrichment analysis (Figure 7B). Based on the abovementioned enrichment results, we speculated that SNHG6 played an important role in the metabolism of CRC.

#### Pathway Enrichment Analysis of Genes Related to SNHG6 Expression in Colorectal Cancer

The LinkedOmics database was used to identify genes that were positively or negatively related to SNHG6 with statistical significance (Figures 7C,D). KEGG and Wikipathway cancer enrichment analyses were conducted in WebGestalt on the genes identified. Oxidative phosphorylation and spliceosome were among the first five terms in the KEGG enrichment analysis (Figure 7E), while metabolic reprogramming in colon cancer was among the top terms identified through the Wikipathway cancer enrichment analysis (Figure 7F), which was consistent with the enrichment results of the ChIRP–MS analysis which found that SNHG6 may play an important role in the metabolism of CRC.

### The SNHG6/HNRNPA1/PKM Axis Was Found to Be Involved in the Metabolic Mechanism of Colorectal Cancer

From the enrichment results of the ChIRP–MS analysis and the SNHG6-related genes identified above, we speculated that SNHG6 may be involved in the metabolic process of CRC by interacting with hnRNP proteins. It was reported that lncRNAs can interact with hnRNP proteins and induce hnRNP to splice and process mRNA in CRC, prostate cancer, and hepatocellular carcinoma (20, 45, 46). Therefore, we wanted to identify the molecule that was involved with SNHG6 and hnRNP protein complexes in CRC metabolism.

#### Pathway Enrichment Analysis of RNAs That Interact Directly With SNHG6

**Figure 8:**
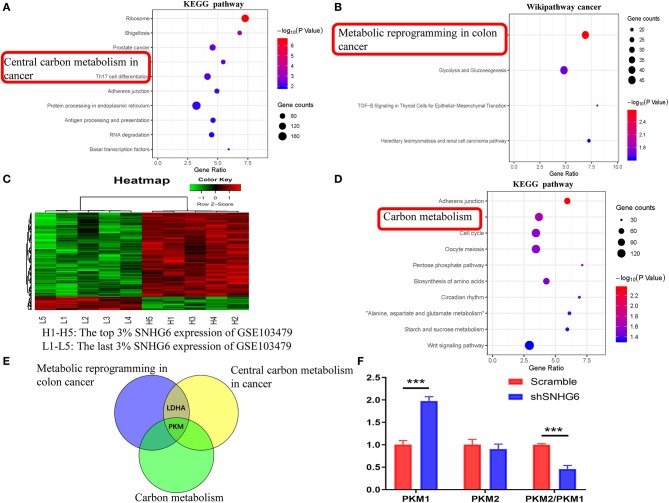
Identification of the involvement of the SNHG6/HNRNPA1/PKM axis in the metabolic mechanism of colorectal cancer (CRC). **(A,B)** Kyoto Encyclopedia of Genes and Genomes (KEGG) and Wikipathway cancer pathway enrichment analysis of mRNA interacting directly with SNHG6, as given by ENCORI. **(C)** A total of 181 differentially expressed genes related to SNHG6 were identified in GSE103479. **(D)** KEGG pathway enrichment analysis of the differentially expressed genes related to SNHG6 as found in GSE103479. **(E)** Through the intersection of genes from metabolic pathways enrichment analyses in the **(A,B,D)**, it was found that pyruvate kinase M (PKM) was the only overlapping gene. **(F)** The ratio of PKM2/PKM1 was downregulated in shSNHG6 RKO cells. Ns, *P* > 0.05; ^*^P < 0.05; ^**^P < 0.01; ^***^P < 0.001; ^****^P < 0.0001.

The interaction between the RNA–SNHG6 pairs was evaluated using ENCORI and 199 RNA molecules, which mainly included protein-coding mRNA, were identified. Then, KEGG and Wikipathway cancer enrichment analyses were conducted in WebGestalt on the genes identified using ENCORI. Central carbon metabolism in cancer was one of the terms which was identified and showed the highest degree of KEGG enrichment (Figure 8A) and likewise with metabolic reprogramming in colon cancer for the Wikipathway cancer enrichment analysis (Figure 8B).

#### Pathway Enrichment Analysis of Genes Correlated With SNHG6 in GSE103479

Based on the SNHG6 expression level in GSE103479, the top 3% and the bottom 3% of samples were grouped as the high SNHG6 group and low SNHG6 group, respectively, and then 181 differentially expressed genes were identified using limma package in R (Figure 8C). WebGestalt was used to explore the KEGG and Wikipathway cancer enrichment based on the identifed differentially expressed genes. Carbon metabolism is one of the terms identified and it showed the highest degree of KEGG enrichment (Figure 8D).

The intersection of genes identified through the metabolic pathways of the three enrichment results given above found that PKM was the only gene that ovelapped (Figure 8E), suggesting that there may be a strong interaction between SNHG6 and PKM. It has been previously reported that hnRNPA1 is involved in the variable splicing of PKM mRNA, the upregulation of the ratio of PKM2/PKM1, and the promotion of cancer through aerobic glycolysis, which can be explained through the Warburg effect (13, 14, 16). As a result, we speculated that SNHG6 could impact the expression of PKM by interacting with hnRNPA1, which is an important mechanism involved in the metabolism of CRC.

### SNHG6 Expression Is Positively Correlated With PKM2/PKM1 Ratio

In RKO cell lines, after knocking down the expression of SNHG6, the qRT-PCR results showed that the ratio of PKM2/PKM1 was downregulated, suggesting that there was a positive correlation between SNHG6 and the ratio of PKM2/PKM1 (Figure 8F).

### Discussion on SNHG6/hnRNPA1/PKM Interaction

#### The Binding Site of SNHG6 Interacting With hnRNPA1

**Figure 9:**
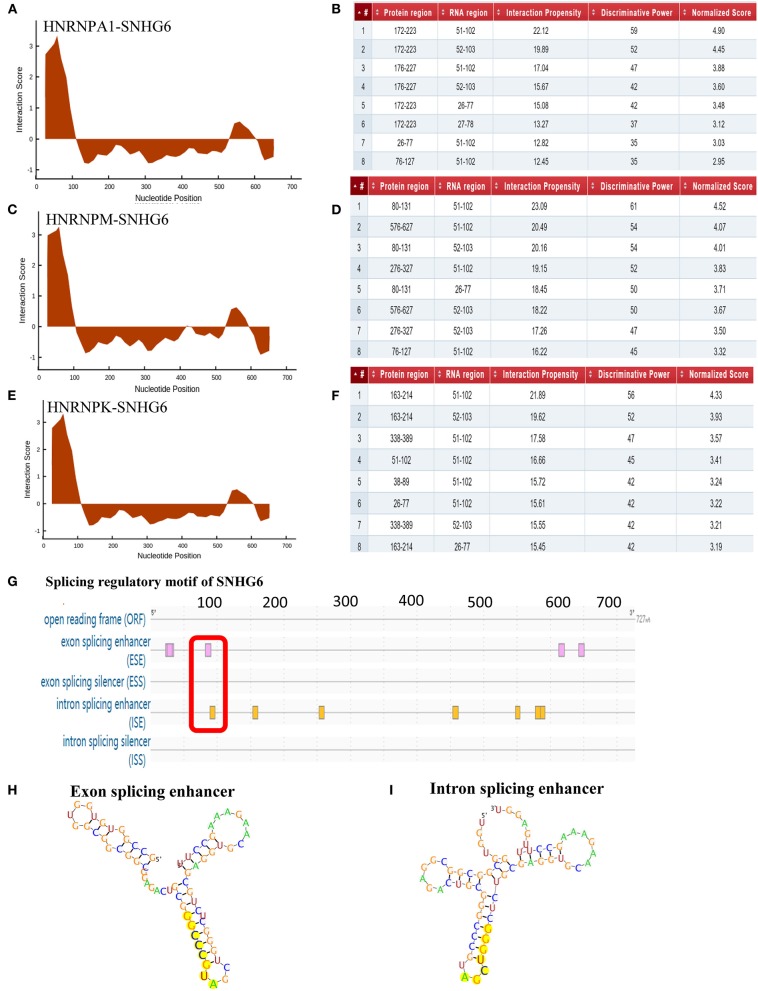
Analysis of binding sites between SNHG6 and hnRNPA1. **(A–F)** The nucleotides at position 51–102 of SNHG6 were in the main region that interacted with hnRNPA1, hnRNPM, and hnRNPK. The amino acids at positions 172–223, 26–77, and 76–127 of hnRNPA1 were in the main regions binding to the nucleotides at position 51–102 of SNHG6. **(G)** An exon enhancer was identified at position 83–90 and an intron enhancer was identified at position 9096 of SNHG6. **(H)** RNA secondary structure of the exon enhancer of SNHG6 at position 83–90. **(I)** RNA secondary structure of the intron enhancer of SNHG6 at position 90–96.

CatRAPID was used to predict the binding site of SNHG6 that interacted with hnRNPA1. It was shown that nucleotides at position 51–102 of SNHG6 constituted the main region that interacted with hnRNPA1 (Figures 9A,B). The amino acids at position 172–223, 26–77, and 76–127 of hnRNPA1 were the main regions that bound with the nucleotides at position 51–102 of SNHG6 (Figures 9A,B). The nucleotides at position 51–102 of SNHG6 were also involved in the interaction between hnRNPK and hnRNPM (Figures 9C–F).

#### Splicing Regulatory Motif of SNHG6 Was Associated With hnRNPA1

RegRNA 2.0 was used to predict the splicing regulatory motif of SNHG6, and two splicing motifs were identified at nucleotide position 51–102. An exon enhancer was identified at position 83–90 and an intron enhancer was identified at position 90–96 (Figures 9G–I), which may be an important splicing motif of SNHG6 to process PKM mRNA by interacting with hnRNPA1.

#### Functional Domains of hnRNPA1 Were Associated With SNHG6

**Figure 10:**
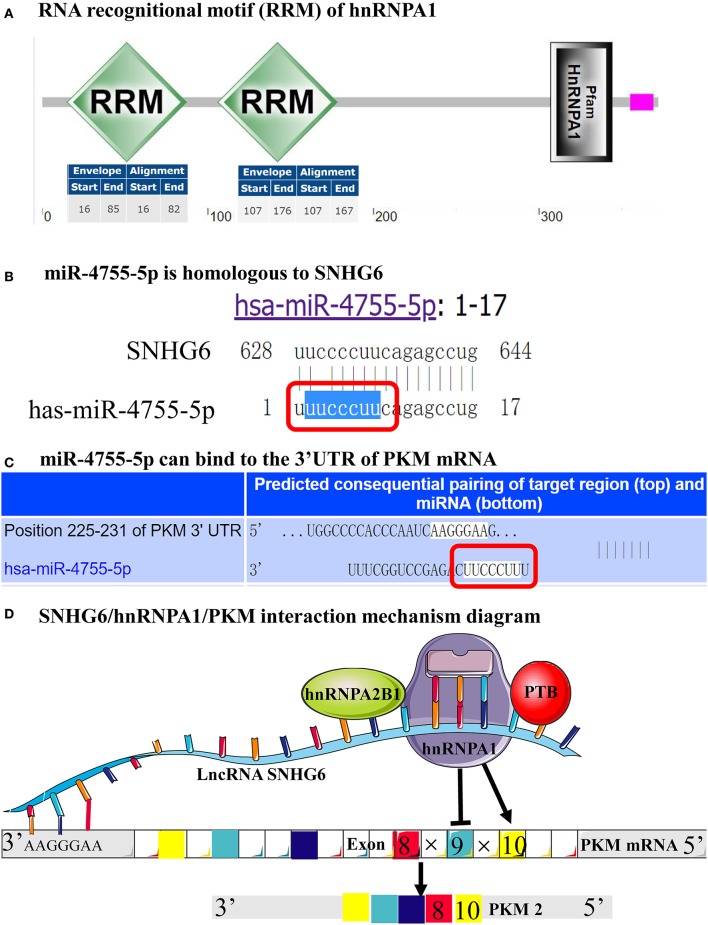
Functional domains of hnRNPA1 and SNHG6 could target the 3′UTR of the pyruvate kinase M (PKM) mRNA. **(A)** The first RNA recognitional motif (RRM) of hnRNPA1 began from the amino acid at position 16 and ended at the amino acid at position 85, while the second RRM of hnRNPA1 began from the amino acid at position 107 and ended at the amino acid at position 176. **(B)** miR-4755-5p was found to be homologous to SNHG6. **(C)** PKM was found to be the target gene of miR-4755-5p. **(D)** Schematic model of SNHG6-mediated aerobic glycolysis which proceeded through the splicing of PKM1/2 as a result of its interaction with the hnRNPA1 complex.

Pfam and SMART were used to identify the functional domains of hnRNPA1 associated with SNHG6, and two RNA recognitional motifs (RRMs) were found in both databases. The first RRM was from the amino acid at position 16 to position 85 and the second from position 107 to position 176, both of which were within amino acids at position 26–223 and therefore might be important domains for the interaction with SNHG6 at nucleotide position 51–102 (Figure 10A).

#### SNHG6 Could Target the 3′UTR of PKM mRNA

The MiRBase database was used to identify microRNAs homologous to SNHG6, and miR-4755-5p was identified (Figure 10B). Then, PKM was found to be a target gene of miR-4755-5p through TargetScanHuman (Figure 10C). The nucleotide sequence of miR-4755-5p binding to the 3′UTR of PKM mRNA was homologous to that of SNHG6 (Figures 10B,C), which indicated that SNHG6 could also target the 3′UTR of PKM mRNA.

## Discussion

Based on GLOBOCAN estimates, about 1.8 million new CRC cases and 0.9 million deaths were recorded worldwide in 2018 (47). Due to the lack of early symptoms and the limitations of timely screening methods, many patients with CRC are diagnosed at an advanced stage (48). At present, the therapeutic effect of advanced CRC is limited to that which is necessary to explore the mechanism of occurrence and the development of CRC. There is growing evidence that lncRNAs are important regulatory molecules involved in a variety of physiological and pathological cellular processes of various cancers (49–51). A large quantity of evidence has revealed that the aberrant expression of lncRNAs may impact the development of CRC by regulating the biological functions of colorectal cancer cells, including proliferation, apoptosis, invasion, and metastasis (52–55).

In our study, we found that the expression of SNHG6 in CRC was high compared with that in many other tumors, while the expression of SNHG6 in CRC was higher than that in heterogeneous colon tissue. In CRC patients, the high expression of SNHG6 was found to be associated with poor prognosis. It has been previously reported that SNHG6 could promote the growth, migration, and invasion of CRC cells *in vitro* and *in vivo* (6, 9, 10). However, research on the carcinogenic mechanism of SNHG6 in CRC has yielded an insufficient result at present, and most research on this mechanism have mainly focused on the carcinogenic role played by SNHG6 by competing as an endogenous RNA to sponge microRNAs (6, 9, 18). In fact, lncRNAs can be used as a scaffold to bring proteins into a complex (19) or as a guide to recruit proteins to process mRNAs (20, 21). As a result, we conducted a ChIRP–MS analysis to identify proteins that interacted with SNHG6 to further explore the carcinogenic mechanism of SNHG6 in CRC.

Through the ChIRP–MS analysis, we identified 467 proteins that could interact with SNHG6 and then used these proteins to construct a PPI network. An important module containing 44 proteins, which could promote invasion, metastasis, and EMT in CRC, was identified in the PPI network. Through the GO and KEGG analysis of the module, it was found that one of the main functions of the module was to splice and process mRNA, which could diversify the carcinogenic function of the module. After the analysis of the proteins that constituted the module, it was found that they were mainly composed of proteins involved in the splicing of mRNA, among which the hnRNP protein family was one of the main components, with six hnRNP proteins included in the top 10 proteins that interacted most strongly with SNHG6. Among the hnRNP proteins, hnRNPM, hnRNPK, and hnRNPA1 were found to be not only positively correlated with the expression of SNHG6 in CRC but also highly expressed in CRC compared with normal tissues. However, the low expression of hnRNPM, hnRNPK, and hnRNPA1 was associated with poor prognosis, which was confusing. Although hnRNPA1 was highly expressed in ovarian cancer tissue compared with normal tissues (Figures S1A,B), it has been reported that hnRNPA1 showed a low expression in drug-resistant ovarian cancer cells (56). Therefore, the low expression of hnRNPA1 associated with the poor prognosis of patients was found to be consistent with the results of the Kaplan–Meier plotter database and The Human Protein Atlas (Figures S1C,D). Therefore, although the expression of hnRNPA1 in CRC was higher than that of normal tissues, it could also promote the development of CRC (57–59). We speculated that its expression was low in drug-resistant CRC strains, which was proven using GSE11440 and GSE118490. However, this mechanism needs to be explored further.

At present, some studies have suggested that lncRNAs can promote cancers, including CRC, through various mechanisms, including by interaction with hnRNP proteins (20). After the above analysis was conducted, we speculated that the specific splicing of mRNA by the complex of SNHG6 and hnRNP proteins may be an important carcinogenic mechanism of SNHG6 in CRC. Through the enrichment analysis, we found that SNHG6 may promote CRC mainly by affecting the metabolic pathways. Genes from the enriched metabolic pathways were intersected, and only PKM, an important molecule for glycolysis metabolism of cancer, was found to be overlapped. Considering that hnRNPA1 can perform the variable splicing of PKM mRNA in order to increase the ratio of PKM2/PKM1 to promote cancer (13, 14, 16), the interaction with hnRNPA1 to splice the mRNA of PKM may be an important mechanism of SNHG6 that is involved in the metabolic process of CRC.

In order to further explore the interaction between SNHG6, hnRNPA1, and PKM, we found that nucleotides at position 51–102 of SNHG6 were the most important sequence for binding to hnRNPA1, which was also the main site of binding to hnRNPM and hnRNPK. Through the motif analysis of the splicing regulation of SNHG6, exactly one exon and one intron were found in the splicing region within nucleotides at position 51 and 102. hnRNPA1 mainly interacted with SNHG6 through the amino acid sequence at position 26 and 223. Then, the functional domain of hnRNPA1 was analyzed, and it was found that there were two RRMs within this sequence. Previous studies have reported that the lncRNAs that interact with the hnRNP protein could target the 3′UTR or the 5′UTR of a specific mRNA to process the mRNA (45, 60). Through the identification of the target gene of miR-4755-5p, which was highly homologous with SNHG6, we found that SNHG6 could target the nucleotide sequence at positon 225–231 of the 3′UTR of the PKM precursor mRNA. Then, we reached the conclusion that by interacting with hnRNPA1, SNHG6 specifically targeted the 3′UTR of PKM mRNA and induced hnRNPA1 to splice the mRNA.

There are two types of PKM: PKM1 and PKM2. PKM2-mediated aerobic glycolysis was found to be more suitable for the occurrence and the development of tumors, and the proportion of PKM2/PKM1 was found to be upregulated in cancer. It has been reported that the variable splicing of exons of PKM precursor mRNA by hnRNPA1, hnRNPA2B1, and polypyrimidine tract binding protein (PTB) complexes is the main mechanism that induces the upregulation of PKM2 expression for the binding of the hnRNPA1 complexes to the splice sites flanking exon 9 in the PKM transcripts, which results in the exclusion of exon 9 and inclusion of exon 10 to generate PKM2 (13, 14, 16). Based on the module identified through the ChIRP–MS analysis, in addition to hnRNPA1, SNHG6 could also interact with hnRNPA2B1 and PTB, suggesting that SNHG6 may be involved in the variable splicing of PKM through the hnRNPA1 protein complex in order to increase the proportion of PKM2/PKM1 (Figure 10D), which was also proven through qRT-PCR results. The upregulation of the PKM2/PKM1 ratio may increase the levels of aerobic glycolysis in CRC cells, which could provide more favorable external conditions for the occurrence and the development of CRC.

In this study, through the results of a joint analysis conducted on SNHG6 using ChIRP–MS and bioinformatics methods, it was found that SNHG6 could interact with hnRNPA1 and specifically target the 3′UTR of the PKM precursor mRNA as well as induce hnRNPA1 to specifically splice the PKM precursor mRNA, which resulted in an increase of the ratio of PKM2/PKM1, which in turn enhanced the aerobic glycolysis process of CRC cells and promoted the development of CRC (Figure 10D). This study is the first to propose the mechanism by which SNHG6 influences the glucose metabolism in CRC. However, the results of the study need to be further verified by conducting further experiments.

## Conclusions

SNHG6 was found to be able to target the 3′UTR of the precursor mRNA of PKM as well as induce the hnRNPA1 protein complex to specifically splice the PKM mRNA. This caused an increase in the proportion of PKM2/PKM1 (Figure 10D), which may be an important mechanism of aerobic glycolysis in CRC.

## Data Availability Statement

The datasets analyzed for this study can be found in the Gene Expression Omnibus (GEO), UALCAN, Oncomine.

## Author Contributions

ZL and XY conducted the bioinformatics analysis included in this study. ZL and XW were responsible for the experimental section. ZL and KS wrote the manuscript. AL checked the manuscript. XW and SL designed the study.

### Conflict of Interest

The authors declare that the research was conducted in the absence of any commercial or financial relationships that could be construed as a potential conflict of interest.

## Supplementary Material

Figure S1 Expression and prognostic values of SNHG6 in Ovarian cancer. **(A,B)** High expression of hnRNPA1 in ovarian cancer. **(C,D)** Low expression of hnRNPA1 was associated with poor prognosis in ovarian cancer. Ns, *P* > 0.05; ^*^*P* < 0.05; ^**^*P* < 0.01; ^***^*P* < 0.001; ^****^*P* < 0.0001.
