# Supplementary figures and images for: The Interaction Between lncRNA SNHG6 and hnRNPA1 Contributes to the Growth of Colorectal Cancer by Enhancing Aerobic Glycolysis Through the Regulation of Alternative Splicing of PKM
Source: Front Oncol. 2020 Mar 31;10:363. doi: 10.3389/fonc.2020.00363 (PMC7136466; doi:10.3389/fonc.2020.00363)

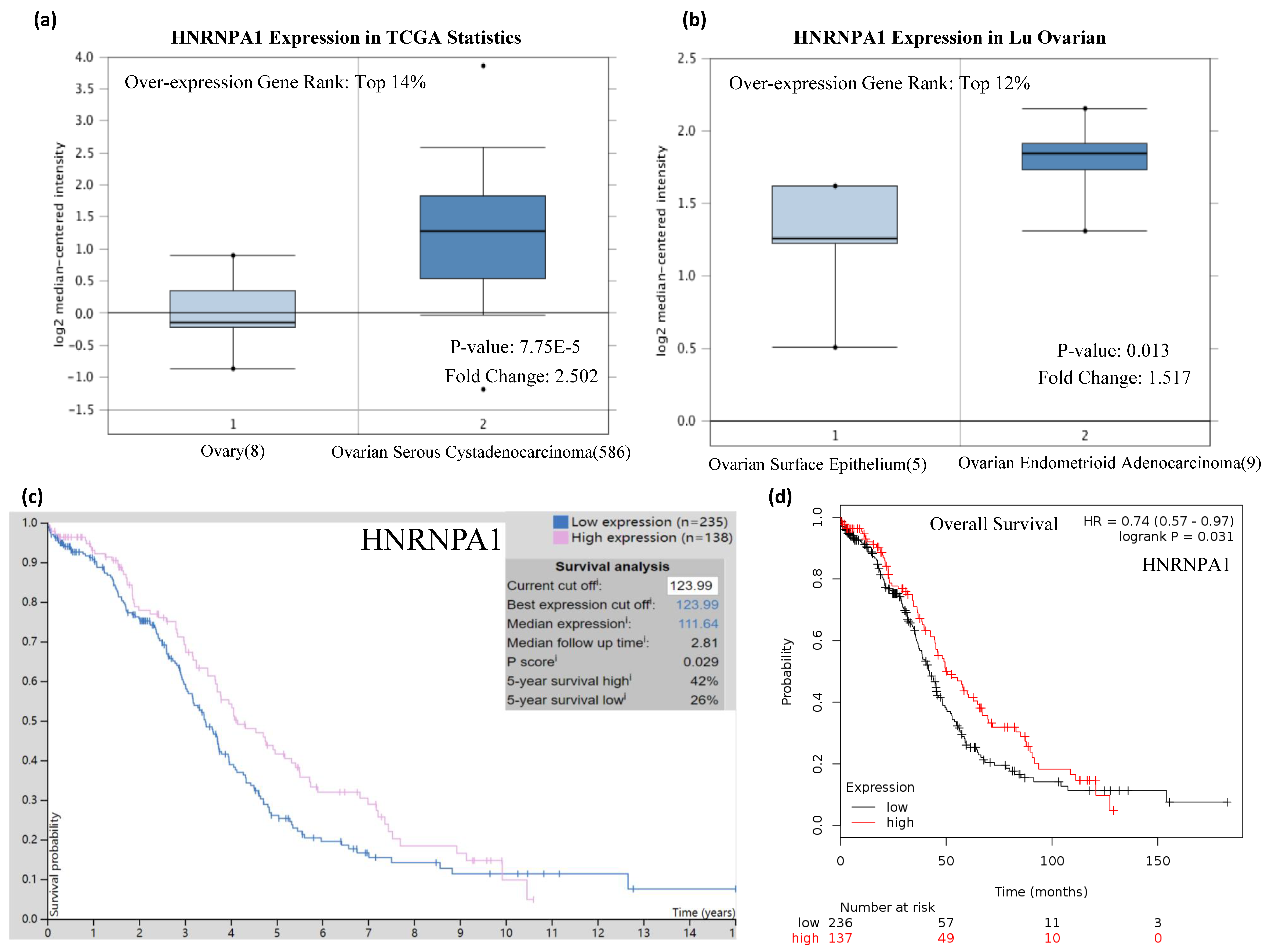

Supplement: Figure S1 — Expression and prognostic values of SNHG6 in Ovarian cancer. (A,B) High expression of hnRNPA1 in ovarian cancer. (C,D) Low expression of hnRNPA1 was associated with poor prognosis in ovarian cancer. Ns, P > 0.05; *P < 0.05; **P < 0.01; ***P < 0.001; ****P < 0.0001. [file Image_1.TIF]
