# Supplementary material for: The Interaction Between lncRNA SNHG6 and hnRNPA1 Contributes to the Growth of Colorectal Cancer by Enhancing Aerobic Glycolysis Through the Regulation of Alternative Splicing of PKM
Source: Front Oncol. 2020 Mar 31;10:363. doi: 10.3389/fonc.2020.00363 (PMC7136466; doi:10.3389/fonc.2020.00363)
Supplement: Supplementary file 2 [file Data_Sheet_1.docx]

**ChIRP-MS analysis method**

1. **Cell harvesting, lysis, disruption**

A total of 2×10^7^ cells were rinsed twice with cold PBS, and digested with trypsin solution. Then cells were collected in a tube at 800 g×4 min, rinsed using 20 ml of PBS, gently shaken up and down, resuspended, centrifuged again at 800 g×4 min. At the end, PBS was removed and cells were stored at -80 ° C。

**2. ChIRP**

Cells were resuspended with PBS and cross-linked with 3% formaldehyde at room temperature (RT) on for 30min. Next, crosslinking was reduced with 125mM glycine for 5min and cells were spun at 1000RCF for 3min. Supernatant was removed and pellets were rinsed twice using PBS.1mL Lysis buffer/2 × 10^7^ cells was added and cell lysates were sonicated in an ice-water bath and checked every 10min until the cell lysate was no longer turbid. They were spun again at high speed and supernatant was transferred into a tube containing 2 volume of Hybridization Buffer. The tubes were incubated at 37 °C. Next, the probe (4 for TT, 1 for NC and PC, 100pmol per 2 × 10^7^ cells) was pre-bound to streptavidin beads for 30min, and the unbound probe was washed out, and mixed with cell lysate, hybridized at 37 °C overnight. Beads were washed 5 times with 1mL pre-warming Wash Buffer. In the end, 1/20 beads were transferred for qPCR investigation. 100μL Elution Buffer and 20U Benzonase was added and protein was eluted at 37 °C for 1h. Supernatant was added to new tube. Beads were washed with 100μL Elution buffer once, and 2 supernatants were combined. Cross-linked sample was reversed at 95 °C, protein was precipitated with 0.1% SDC and 10% TCA at 4 °C 2h. It was spun at top speed and pellets were washed with cold 80% acetone thrice.

**3. Tryptic digestion**

5mM TCEP was added to every sample, incubated and mixed at 55°C for 10 min. 10mM IAA was added once the samples were cooled down to RT, incubated in the dark for 15 min. Trypsin was resuspended with re-suspension buffer to 0.5μg/μL and incubated at RT for 5 min. μL trypsin solution was added to every sample. After proper mixing, samples were spun down and incubated at 37°C for 8 h or overnight. Quenching of the reaction was done with 1% TFA.

**4. Peptide desalting for LC-MS/MS**

C18 column was equilibrated with 200 μL ACN. ACN was washed out with 200μL 0.1% FA 2 times. Peptide solution was loaded to C18 tip column and the solution was allowed to flow through the column slowly, and flow through (A) was collected. The peptide-loading step was repeated one more time. Column was washed with 200μL 0.1% FA. Peptide was eluted with 50μL 70% can and elution (B) was collected. The desalting step was repeated one more time with flow through (A). Two elution (B) were merged and vacuum dried at 4 °C or RT. Peptide was resuspended with 10μL 0.1% FA for LC-MS/MS analysis.

**5. LC-MS/MS**

For each sample, 1/2 peptide were alienated and investigated with a nano-UPLC (EASY-nLC1200) connected to Q-Exactive mass spectrometry (Thermo Finnigan). Separation was accomplished using a reversed-phase column (100 μm, ID × 15 cm, Reprosil-Pur 120 C18-AQ, 1.9μm, Dr. Math). Mobile phases were H_2_O with 0.1 % FA, 2 % ACN (phase A) and 80 % ACN, 0.1 % FA (phase B). Separation of sample was achieved with a 120 min gradient at 300 nL/min flow rate. Gradient B: 8 to 30 % for 92 min, 30 to 40 % for 20 min, 40 to 100 % for 2 min, 100 % for 2 min, 100 to 2 % for 2 min and 2 % for 2 min.

Data dependent acquisition was accomplished in profile as well as positive mode with Orbitrap analyzer at a resolution of 70,000 (@200 m/z) and m/z range of 350-1600 for MS1. For MS2, the resolution was fixed to 17,500. The automatic gain control (AGC) target for MS1 was set to 1.0 E+6 with max IT 100ms. And 5.0 E+4 for MS2 with max IT 200ms. The top 10 furthermost strong ions were disintegrated by HCD with normalized collision energy (NCE) of 27 %, and isolation window of 2 m/z. The dynamic exclusion time window was 20s.

**6. MaxQuant database search**

Raw MS files were managed with MaxQuant (Version 1.5.6.0). The protein sequence database (Uniprot_organism_2016_09) was acquired from UNIPROT. These databases as well as its reverse decoy were then investigated against by MaxQuant software. Trypsin was established as precise enzyme with up to 3 miss cleavage; Oxidation [M] as well as Acetyl [protein N-term] were regarded as variable alteration. Carbamidomethyl [C] was set as fixed alteration. Both peptide as well as protein FDR must be lower than 0.01. Only exclusive as well as razor peptides were utilized for computation. All the additional parameters were kept as default.

**7. Normalization**

It was described as the ratio of the standardized spectral count of protein i in purification with bait j, Ti,j, to the mean standardized spectral sum of that protein across the negative controls. Ci was computed as F Ci,j = (Ti,j + α)/(Ci + α). The standardized spectral sums were calculated as Ti,j = SCi,j/Nj, where the normalization factor was the sum of over all proteins recognized in the experimentation with bait j, Nj = ∑SCi,j. Likewise, the counts were standardized in every negative-control experimentation x = 1...n, Ci,x = SCi,x/Nx, before the averaged normalized count across all n controls, Ci = 1/n∑Ci,x, was calculated. A small background factor α was added to inhibit division by 0, computed as β/ave(Nx), where ave(Nx) was the average normalization factor across all n negative controls. The parameter β was by default set to 1.

**Result of ChIRP-MS**

To ensure the reliability of the result, negative control group was added, as well as positive control group, which used U1snRNA as special probe to enrich U1snRNA-binding protein.

The FDR of the polypeptides and proteins of this experiment was set at the level of 0.01. Contaminated proteins like keratin were firstly removed. Secondly, quantitative analysis of the test group and negative control group was conducted according to the FCa value and the number of unique peptides.

Protein enrichment classification information for comparison U1–Ctrl was displayed as Venn diagram (Figure a). 3 parts of Venn diagram were corresponding to test enriched (U1), control enriched (Ctrl), and none enriched (U1&Ctrl).

Protein enrichment classification information for comparison Lnc–Ctrl was also displayed as Venn diagram (Figure b). 3 parts of Venn diagram were corresponding to test enriched (Lnc), control enriched (Ctrl), and none enriched (Lnc & Ctrl). A total of 467 proteins were enriched and supposed to bind with SNHG6.


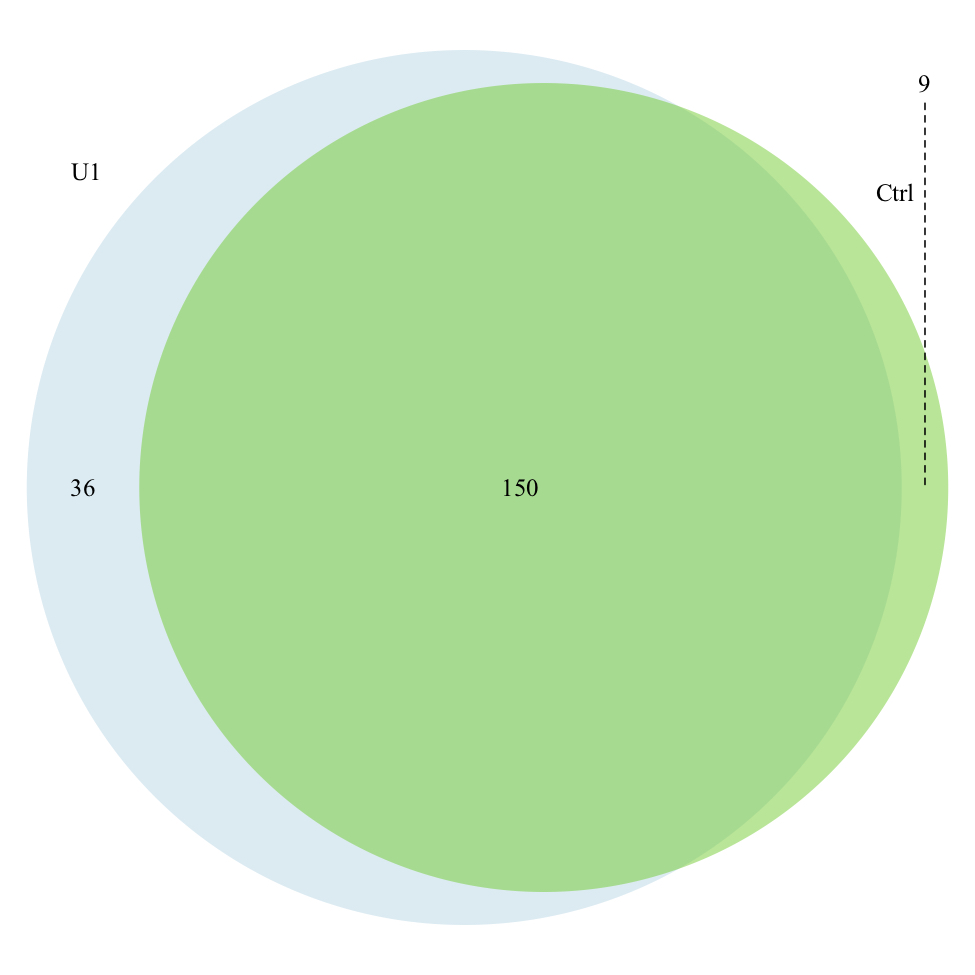


Figure a. Protein enrichment summary of U1 combined proteins with Venn diagram


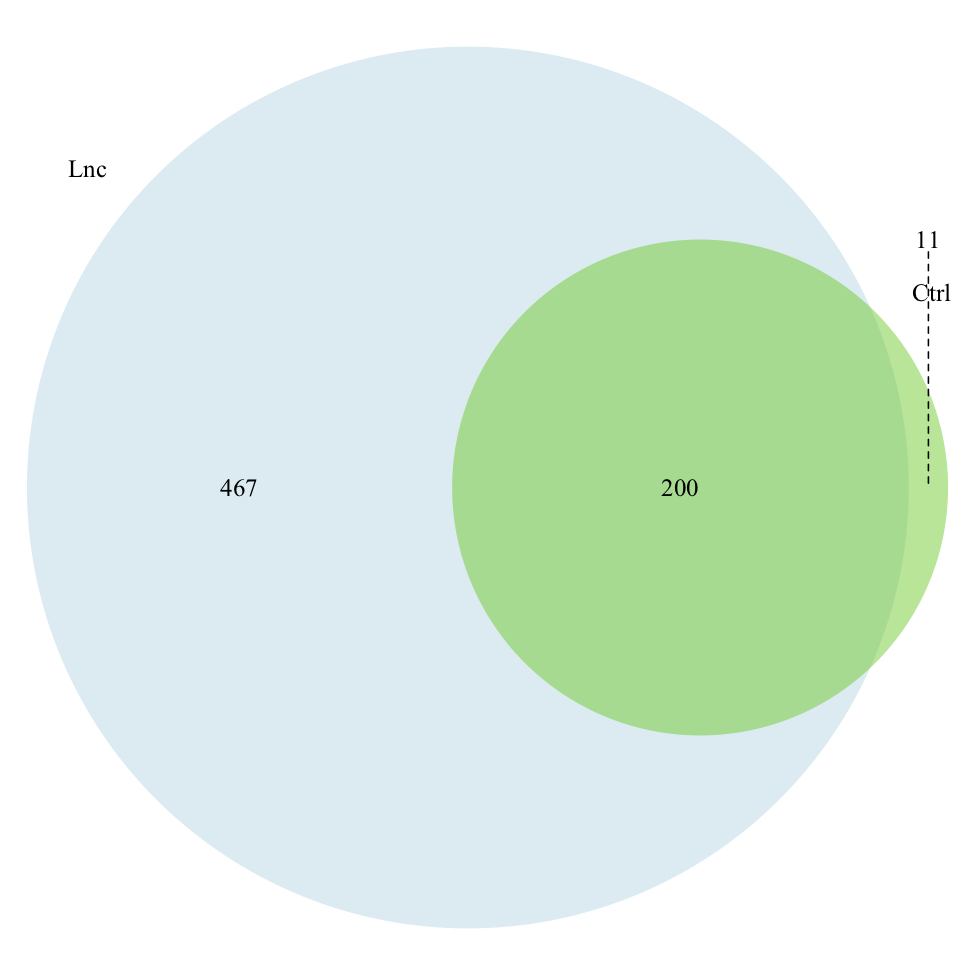


Figure b. Protein enrichment summary of SNHG6 combined proteins with Venn diagram
